# Evidence for aortopathy of the native descending aorta in children with hypoplastic left heart syndrome

**DOI:** 10.1186/1532-429X-17-S1-Q79

**Published:** 2015-02-03

**Authors:** Inga Voges, Michael Jerosch-Herold, Christopher Hart, Dominik Daniel Gabbert, Philip Wegner, Ana C Andrade, Minh H Pham, Ines Kristo, Hans-Heiner Kramer, Carsten Rickers

**Affiliations:** 1 Department of Congenital Heart Disease and Pediatric Cardiology, University Hospital Schleswig-Holstein, Kiel, Germany; 2 Department of Radiology, Brigham & Women's Hospital & Harvard Medical School, Boston, MA, USA; 3 Asklepios Klinik Sankt Augustin GmbH, Sankt Augustin, Germany

## Background

Patients with hypoplastic left heart syndrome (HLHS) after Norwood operation show dilatation and reduced distensibility of the reconstructed proximal aorta. Cardiovascular magnetic resonance imaging and angiographic examinations indicate that also the native descending aorta (DAo) is dilated, but this has not been intensively studied.

## Methods

79 children with HLHS in Fontan circulation (6.3±3.2 years) and 18 controls (6.8±2.4 years) underwent 3.0 Tesla cardiovascular magnetic resonance imaging. Gradient-echo cine and phase-contrast imaging was applied to measure cross-sectional areas, distensibility and pulse wave velocity (PWV) of the entire thoracic aorta, particularly the DAo. Cross-sectional areas were compared with normal values for healthy children.

## Results

Patients had significantly elevated cross-sectional areas of the DAo at different levels vs. controls (DAo, pulmonary artery bifurcation: 229.1±97.2 vs. 175.7±24.3 mm/m^2^; DAo, diaphragm: 196.2±66.0 vs. 142.6±16.7 mm/m^2^; p<0.05), but similar bioelastic properties (p>0.05). In 41 (52%) patients cross-sectional areas of the DAo exceeded the 95^th^ percentile. These HLHS patients showed a higher PWV of the thoracic DAo compared to HLHS patients with normal cross-sectional areas (4.0±1.1 vs. 3.4±1.3 m/s, p<0.05).

## Conclusions

About half of our HLHS patients in this study showed aortic dilatation and increased PWV, both signs of aortopathy of the DAo. Close follow up is warranted to determine potential future clinical implications for these patients.

## Funding

None.

